# Adverse events in orthopedic care identified via the Global Trigger Tool in Sweden – implications on preventable prolonged hospitalizations

**DOI:** 10.1186/s13037-016-0112-y

**Published:** 2016-10-26

**Authors:** Hans Rutberg, Madeleine Borgstedt-Risberg, Pelle Gustafson, Maria Unbeck

**Affiliations:** 1 Department of Medical and Health Sciences, Division of Health Care Analysis, Linköping University, Linköping, Sweden; 2 Swedish Association of Local Authorities and Regions, Stockholm, Sweden; 3 Centre for Healthcare Development, Region Östergötland, Linkoping, Sweden; 4 Department of Clinical Sciences Lund, Orthopedics, Lund University, Skane University Hospital, Lund, Sweden; 5 Department of Clinical Sciences, Danderyd Hospital, Karolinska Institutet, Stockholm, Sweden; 6 Department of Orthopedics, Danderyd Hospital, Stockholm, Sweden; 7 Department of Orthopedics, Skane University Hospital, SE-221 85 Lund, Sweden

**Keywords:** Adverse event, Global Trigger Tool, Retrospective record review, Orthopedic care, Patient safety

## Abstract

**Background:**

The national incidence of adverse events (AEs) in Swedish orthopedic care has never been described. A new national database has made it possible to describe incidence, nature, preventability and consequences of AEs in Swedish orthopedic care.

**Methods:**

We used national data from a structured two-stage record review with a Swedish modification of the Global Trigger Tool. The sample was 4,994 randomly selected orthopedic admissions in 56 hospitals during 2013 and 2014. The AEs were classified according to the Swedish Patient Safety Act into preventable or non-preventable.

**Results:**

At least one AE occurred in 733 (15 %, 95 % CI 13.7–15.7) admissions. Of 950 identified AEs, 697 (73 %) were judged preventable. More than half of the AEs (54 %) were of temporary nature. The most common types of AE were healthcare-associated infections and distended urinary bladder. Patients ≥65 years had more AEs (*p* < 0.001), and were more often affected by pressure ulcer (*p* < 0.001) and urinary tract infections (*p* < 0.01). Distended urinary bladder was seen more frequently in patients aged 18–64 years (*p* = 0.01). Length of stay was twice as long for patients with AEs (*p* < 0.001). We estimate 232,000 extra hospital days due to AEs during these 2 years. The pattern of AEs in orthopedic care was different compared to other hospital specialties.

**Conclusions:**

Using a national database, we found AEs in 15 % of orthopedic admissions. The majority of the AEs was of temporary nature and judged preventable. Our results can be used to guide focused patient safety work.

## Background

The identification and reduction of adverse events (AEs) is of great importance in order to minimize patient suffering. There are several methods to identify AEs [1]. Retrospective record review (RRR) is a commonly used method. It is considered less sensitive to under-reporting than other methods, such as clinical incident reports, patient safety indicators, complaints or medico-legal claims [2–4]. RRR can be repeated over time, and specific AE types can be identified [5].

One of the most commonly used is the Global Trigger Tool (GTT), developed by the Institute for Healthcare Improvement [6]. In 2008, a Swedish translation and adaptation of the GTT method was introduced. A revised version of the method [7] was published in 2012, mainly in order to have a RRR method compliant with a new Swedish Patient Safety Act, but also to incorporate common AEs not included in the original method such as distended urinary bladder, failure in vital signs and neurological injury. A few of the original triggers in the GTT manual were omitted since they were seldomly found and in order to improve the review process the trigger definitions were expanded with more detailed instructions for the judgment of AEs and its preventability.

In 2011, the Swedish government and The Swedish Association of Local Authorities and Regions (SALAR) launched a national initiative to reduce patient harm. As one of several components of this initiative, RRR has been done in all 63 Swedish acute or mixed acute and planned care hospitals since 2012.

Studies indicate that orthopedics is one of the specialties where AEs are most common [8, 9]. The national incidence and pattern of AEs in Swedish orthopedic care have never been described. Therefore, the aim of this study was to describe the incidence, nature, preventability and consequences of AEs in Swedish orthopedic care.

## Methods

### Sample and setting

The RRR was performed as a part of a national patient safety initiative. The RRR included all patients admitted to inpatient somatic healthcare performed by county councils and regions. Data was collected from all 63 Swedish hospitals that provide either acute or mixed acute/planned care. Private units that provide strictly planned orthopedic care were excluded. These elective units are estimated to represent 5 % of all orthopedic care in Sweden. Thus, we estimate that our sample represents 95 % of all orthopedic care provided in Sweden during 2013 and 2014.

**Fig. 1:**
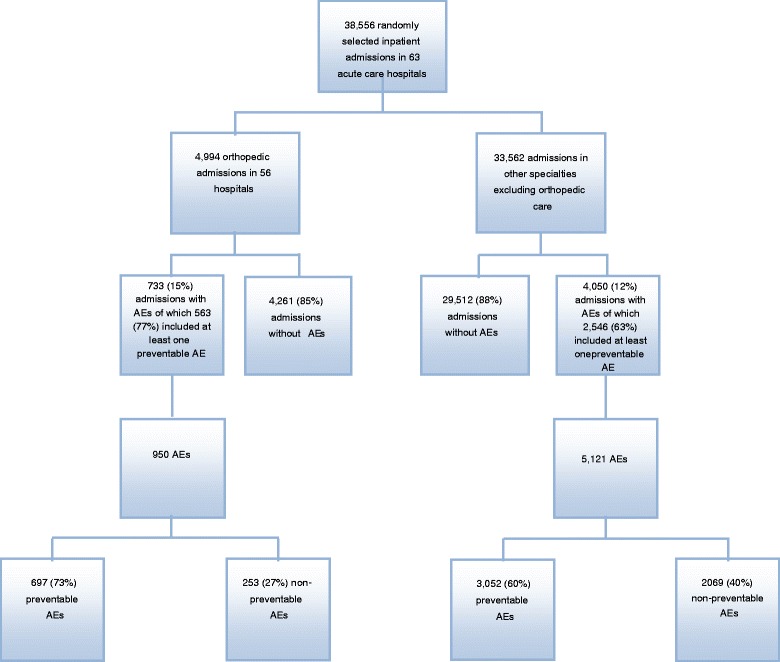
Flow chart of the review process. AEs, adverse events

Records of inpatient admissions of patients ≥18 years who were discharged from 1 January 2013 to 31 December 2014 and with at least a 24-h length of stay were eligible for randomization. The minimum monthly number of admissions for review was 40 for university hospitals, 30 for central county council hospitals and 20 for county council hospitals. During 2013 and 2014, a total of 38,556 random inpatient admissions have been reviewed, of which 4,994 were orthopedic admissions (Fig. 1).

### Definitions and inclusion criteria

In the Swedish handbook [7], an AE is defined as an unintended physical injury resulting from medical care, and that requires additional monitoring, treatment, or hospitalisation, or results in death. A preventable AE is defined as an AE which could have been prevented if adequate actions had been taken during the patient’s contact with healthcare. This definition is based on the terminology in the Swedish Patient Safety Act [10]. Both AEs related to acts of omission and acts of commission are included. We used these definitions in this study.

Orthopedic care was defined as care in, or initiated from, orthopedic wards. We included both admissions with or without surgery. The orthopedic admission constituted the index admission, but if the patient had been treated by other medical specialties (psychiatric admissions excluded) or on other wards during the admission, this care was also included in the index admission.

To be classified as an AE, one of the following three criteria had to be met: (1) the AE occurred within 30 days before the index admission and caused the index admission; (2) the AE occurred and was detected during the index admission; (3) the AE was related to the index admission and was detected within 30 days of discharge. AEs identified by using the latter criterion were not required to result in a new admission; thus an AE treated on an outpatient basis was included.

### Review teams and training

Formal training for the review teams in RRR methodology was given in the beginning of 2012 via SALAR. Follow-up meetings for further discussions on how to use the method, and for introduction of the revised handbook, took place during the autumn of 2012. Team members were all senior professionals with knowledge and experience in the field of patient safety. The review teams consisted of one or two trained registered nurses and at least one physician, representing different medical specialties.

### Review process

First, all randomly selected records were screened for a maximum of 20 min, by one or two members of the review team, for presence of one or more of the 44 defined triggers, each representing a potential AE. If a trigger was identified, the whole team met and a consensus decision was reached whether the potential AE constituted an AE.

The decision to classify the AE as a probably preventable or a preventable AE was made as a consensus decision by the whole team. A 4-grade scale was used, where the grades were: 1 = the AE was not preventable, 2 = the AE was probably not preventable, 3 = the AE was probably preventable, and 4 = the AE was preventable. Due to the difficulties in retrospective classification, and to avoid discussions on clearly vs probably preventable or not, grades 1 and 2 were grouped and contrasted with grades 3 and 4. Henceforth, probably preventable and preventable AEs will be named preventable AEs.

The severity of the AE was judged using an adaptation of the National Coordinating Council for Medication Error Reporting and Prevention Index (NCC MERP) [11]. The NCC MERP Index categories E through I (i.e. those relating to harm) were included. The AEs were also categorized according to the type of AE.

A web-based portal, with access only for the contact person of the respective hospital, was used for entry of anonymized patient and AE data into the national database at SALAR.

### Statistics

Demographic data are presented as mean or median (SD or range). Comparison of proportions between two groups was made by Fisher’s Exact Test. Comparisons of proportions between more than two groups was made by Pearson Chi-Square Test. Confidence intervals was calculated using normal distribution approximation. *P*-values <0.05 was considered statistically significant.

The calculations of hospital days in this study were based on the average number of hospital days for an admission with or without an AE. According to these data the additional number of hospital days has been aggregated to national level based on the total number of admissions in orthopedic care in the national inpatient register at the National Board of Health and Welfare during 2013 and 2014.

All statistical calculations were made by SPSS version 22.

## Results

According to the National Board of Health and Welfare’s inpatient register, the total number of orthopedic admissions in Sweden during 2013 and 2014 was 253,612, of which 185,591 included a surgical procedure. 27 county council hospitals (*n* = 2,488 admissions), 22 central county council hospitals (*n* = 2,113 admissions) and 7 university hospitals (*n* = 393 admissions), in total 56 hospitals of the 63 eligible and 4,994 admissions, were included in the study (Fig. 1).

The majority of the patients were women (*n* = 2,866, 57 %). The mean (median, range) age for women was 72.5 (75, 18–107) years and 66.0 (69, 18–102) years for men. 75 % of the women and 61 % of the men were 65 years or older. The mean (median, range) length of hospital stay (LOS) was 7.0 (5, 1–121) days.

In total, 950 AEs were identified in 733 patients (15 %, 95 % CI 13.7–15.7), range 1–7 AEs per patient. Two or more AEs were identified in 147 admissions. 697 AEs in 563 patients (77 %, 95 % CI 73.8–79.9) were classified as preventable (Table 1). The number of AEs per 1,000 hospital days was 27.0 and the number of AEs per 100 admissions was 19.0. The corresponding numbers for preventable AEs were 19.8 and 14.0.

**Table 1:** Type and number of adverse events (AEs) and preventable^a^ AEs in orthopedic care and other specialties

Type of AE	AEs in orthopedic care n (%)	AEs in other specialties excluding orthopedic care n (%)	Preventable^a^ AEs in orthopedic care n (%)	Preventable^a^ AEs in other specialties excluding orthopedic care n (%)
Healthcare-associated infection	344 (36)	1699 (33)	245 (71)	1030 (61)
Postoperative wound infection	135 (14)	330 (6)	104 (77)	255 (77)
Urinary tract infection	100 (11)	471 (9)	72 (72)	282 (60)
Other infection	44 (5)	432 (8)	26 (59)	230 (53)
Pneumonia (excluding ventilator- associated pneumonia)	32 (3)	197 (4)	17 (53)	106 (54)
Sepsis	14 (1)	157 (3)	9 (64)	87 (55)
Ventilator-associated pneumonia	8 (1)	27 (1)	7 (88)	20 (74)
Central line associated infection	7 (1)	37 (1)	7 (100)	29 (78)
Clostridium difficile positive stool	4	48 (1)	3 (75)	21 (44)
Distended urinary bladder	136 (14)	421 (8)	121 (89)	373 (89)
Surgical and other invasive AEs	130 (14)	655 (13)	82 (63)	374 (57)
Reoperation	51 (5)	181 (4)	35 (69)	121 (67)
Other surgical AEs	36 (4)	247 (5)	20 (56)	136 (55)
Postoperative hemorrhage or hematoma (not requiring reoperation)	34 (4)	138 (3)	20 (59)	64 (46)
Organ injury	7 (1)	88 (2)	5 (71)	52 (59)
Wrong side, wrong site, wrong patient	2	1	2 (100)	1 (100,0)
Pressure ulcer (category 2–4)	89 (9)	342 (7)	85 (96)	303 (89)
Adverse drug event	59 (6)	512 (10)	41 (69)	239 (47)
Other AEs	48 (5)	302 (6)	34 (71)	139 (46)
Skin or superficial vessel AEs	40 (4)	312 (6)	34 (85)	236 (76)
Falls	35 (4)	297 (6)	29 (83)	168 (57)
Thrombosis or emboli	21 (2)	93 (2)	4 (19)	37 (40)
Failure in vital signs	18 (2)	138 (3)	8 (44)	55 (40)
Hemorrhage not connected to surgery	14 (1)	123 (2)	7 (50)	27 (22)
Neurological injury	6 (1)	23	4 (67)	7 (30)
Allergic reaction	6 (1)	74 (1)	0	14 (19)
Anesthesia related AEs	4	30 (1)	3 (75)	16 (53)
Medical technique related AEs	0	10	0	3 (30)
Postpartum or obstetric AEs	0	90 (2)	0	31 (34)
Total	950 (100)	5121 (100)	697 (73)	3052 (60)

^a^including both probably preventable and preventable AEs

The table is sorted on the column AEs in orthopaedic care n (%)

Patients ≥65 years of age had more admissions with at least one AE compared to younger patients, 17 % vs. 10 % (*p* < 0.001). The incidence of preventable AEs was higher in patients ≥65 years old, 13 % vs. 8 % (*p* < 0.001). No difference was found in the incidence of AEs in men and in women, 15 % vs. 15 % (*p* = 0.87).

The incidence of AEs between hospitals ranged from 3 to 43 %. Concerning hospital type; county council hospitals had a significantly lower incidence of AEs compared to central county council and university hospitals (11, 18 and 19 %, respectively, *p* < 0.001). This pattern was also seen in the preventable AEs (8, 14 and 15 %, respectively, *p* < 0.001).

### The nature and consequences of adverse events

Healthcare-associated infection was the most common type of AE (36 %), followed by distended urinary bladder and surgical and other invasive AEs (Table 1). Among infections, postoperative wound infections and urinary tract infections were the most common. AEs in orthopedic care related to central line associated infections and wrong site/side/patient surgery were judged to be preventable in all cases, followed by pressure ulcers and distended urinary bladders (96 % vs. 89 % preventability) (Table 1).

Distended urinary bladder was found to be more frequent among patients 18 to 64 years, *p* = 0.01). Pressure ulcers and urinary tract infections were more common in patients aged 65 or more, *p* < 0.001 and *p* < 0.01.

Half of the AEs were of temporary and of less severe nature. AEs leading to increased LOS, readmissions or outpatient visits occurred in 42 % of all AEs (Table 2). The five AEs that contributed to the death of the patient occurred in patients ≥65 years. The percentage of AEs that resulted in permanent harm was higher for younger patients (18–64 years) than for older patients. All of these AEs in younger patients were considered preventable.

**Table 2:** Severity of adverse events (AEs) and preventable^a^ AEs in orthopedic care and other specialties

Severity category	AEs in orthopedic care n (%)	AEs in other specialties excluding orthopedic care n (%)	Preventable^a^ AEs in orthopedic care n (%)	Preventable^a^ AEs in other specialties excluding orthopedic care n (%)
Category E	509 (54)	2557 (50)	385 (76)	1553 (61)
Category F	401 (42)	2306 (45)	283 (71)	1369 (59)
Category G	30 (3)	116 (2)	24 (80)	63 (54)
Category H	5 (1)	30 (1)	3 (60)	19 (63)
Category I	5 (1)	112 (2)	2 (40)	64 (57)
Total	950 (100)	5121 (100)	697 (73)	3052 (60)

^a^including both probably preventable and preventable AEs

*E* contributed to or gave temporary harm that needed intervention, *F* contributed to or gave temporary harm and required outpatient care, hospital care or prolonged hospital stay, *G* contributed to or gave permanent patient harm, *H* lifesaving intervention required within 60 min, *I* contributed to patient’s death

The table is sorted on the column AEs in orthopaedic care *n* (%)

More than half of the AEs concerning anesthesia related AEs, thrombosis or emboli, surgical and other invasive AEs, and healthcare-associated infections led to increased LOS, readmissions or outpatient visits (75, 71, 60 and 52 %, respectively) (Table 3). No differences could be found concerning age and the respective severity category (Category E-I, *p* = 0.08, 1.00, 0.48, 1.00, 0.59, respectively).

**Table 3:** Number of adverse events (AEs) grouped in type of AEs according to severity of harm

Type of AE	Category E n (%)	Category F n (%)	Category G n (%)	Category H n (%)	Category I n (%)
Healthcare-associated infection	151 (44)	180 (52)	10 (3)	1	2 (1)
Urinary tract infection	78 (78)	22 (22)	0	0	0
Postoperative wound infection	28 (21)	99 (73)	7 (5)	0	1 (1)
Other infection	25 (57)	17 (39)	2 (5)	0	0
Pneumonia (excluding ventilator-associated pneumonia)	7 (22)	23 (72)	0	1 (3)	1 (3)
Central line associated infection	4 (57)	3 (43)	0	0	0
Ventilator-associated pneumonia	4 (50)	4 (50)	0	0	0
Clostridium difficile positive stool	3 (75)	1 (25)	0	0	0
Sepsis	2 (14)	11 (79)	1 (7)	0	0
Distended urinary bladder	123 (90)	12 (9)	1 (1)	0	0
Pressure ulcer (category 2–4)	67 (75)	22 (25)	0	0	0
Surgical and other invasive AEs	41 (32)	78 (60)	10 (8)	1 (1)	0
Other surgical AEs	17 (47)	15 (42)	3 (8)	1 (3)	0
Postoperative hemorrhage or hematoma (not requiring reoperation)	16 (47)	18 (53)	0	0	0
Reoperation	7 (14)	40 (78)	4 (8)	0	0
Wrong side, wrong site, wrong patient	1 (50)	1 (50)	0	0	0
Organ injury	0	4 (57)	3 (43)	0	0
Adverse drug event	32 (54)	26 (44)	0	1 (2)	0
Skin or superficial vessel AEs	31 (78)	9 (23)	0	0	0
Falls	23 (66)	10 (29)	2 (6)	0	0
Other AEs	22 (46)	25 (52)	1 (2)	0	0
Hemorrhage not connected to surgery	6 (43)	7 (50)	1 (7)	0	0
Thrombosis or emboli	5 (24)	15 (71)	1 (5)	0	0
Allergic reaction	4 (67)	2 (33)	0	0	0
Failure in vital signs	3 (17)	9 (50)	1 (6)	2 (11)	3 (17)
Anesthesia related AEs	1 (25)	3 (75)	0	0	0
Neurological injury	0	3 (50)	3 (50)	0	0
Total	509 (54)	401 (42)	30 (3)	5 (1)	5 (1)

*E* contributed to or gave temporary harm that needed intervention, *F* contributed to or gave temporary harm and required outpatient care, hospital care or prolonged hospital stay, *G* contributed to or gave permanent patient harm, *H* lifesaving intervention required within 60 min, *I* contributed to patient’s death

The table is sorted on the column Category E *n* (%)

### Length of hospital stay

**Fig. 2:**
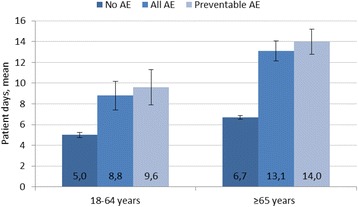
Length of stay for orthopedic admissions without adverse events (AEs), with AEs and with at least one preventable* AE grouped according to age group. * including both probably preventable and preventable AEs. AE, Adverse event

The mean LOS was 12.2 (SD 11.4) days for patients with an AE, compared to 6.1 (SD 4.7) days in patients without any AE. The corresponding for patients affected by a preventable AE was 13.1 (SD 12.2) days. LOS was longer in both older and younger patient groups for admissions with AEs. When comparing older and younger patients with an AE or at least one preventable AE, older patients had a longer LOS (Fig. 2).

Based on an AE incidence of 15 %, and a mean increase of LOS of 6.1 days for patients with an AE during primary stay, the aggregated total number of extra hospital days due to AEs was estimated to 116,000 days per year. This can also be expressed that every day, around 300 Swedish orthopedic beds are occupied because of an AE, of which 73 % are preventable.

### Comparison of orthopedic care with other specialties

The AE incidence in other inpatient specialties included in the review process was 12 % (95 % CI, 11.7–12.4). The incidences of both unpreventable and preventable AEs were higher in orthopedic care (*p* < 0.001). Distended urinary bladder and pressure ulcers were more common in orthopedics while, for example, adverse drug events and AEs related to falls were less common compared to other specialties. Half of the AEs were of temporary and of less severe nature, both in orthopedics and in other specialties (50 % vs. 54 %). AEs contributing to the death of the patient were more frequent in other specialties (Tables 1, 2 and 3).

## Discussion

To our knowledge, this is the largest national study concerning AEs in orthopedic care using a structured RRR method. We found an AE incidence of 15 %. The most common AE type was hospital-associated infections, followed by distended urinary bladder and AEs related to surgical procedures. More than half of the AEs were minor and of temporary nature. LOS increased in the AE group. Patients ≥65 years were more often affected by an AE than younger patients.

The nature and incidence of AEs differ according to specialty and procedures utilized within different specialties [8, 12–14]. Surgical specialties often have higher incidence of AEs compared to non-surgical specialties [9, 15, 16]. Whether AEs in surgical care occur more frequently or simply are more often detected by patients and healthcare personnel, is not known. Greater treatment complexity and invasiveness of care compared to medical specialties may increase the risk for an AE. An AE incidence of 15 % can be regarded as high, but earlier studies have shown an AE incidence up to 30 % in orthopedic care [14, 17, 18]. A review [19] reported that 14 % of surgical patients were affected by AEs. It is reasonable to assume that, to prevent AE, specialty-specific type and incidence must be presented.

The judgment of AEs and preventability can be difficult, which is illustrated by our finding that more than 60 % of preventable AEs were classified as probably preventable. Many AEs are traditionally considered as unpreventable complications, but a number of these may be defined at a post-audit as preventable. In the RRR methodology used, the reviewers were encouraged to judge the findings from the patient’s view and in this way complications and AEs were merged and reported as a whole. In our study, 11 % of the patients were affected by a preventable AE. Other surgical RRR studies, using different RRR methods, reported preventable AEs in 3–11 % of the patients [19].

The most common AE type was healthcare-associated infections. This type of AE was also found to be common in other specialties in this study, and in other studies [14, 15, 20]. More than one-third of the healthcare-associated infections were postoperative wound infections. The Swedish National Patient Insurance Company has since 2009 worked together with six professional organizations with a nationwide project named Prosthesis-related infections shall be stopped (PRISS) with the overall aim to reduce the frequency of prosthesis related infection after primary hips and knee surgeries with 50 % [21]. The project has shown that interventions are needed on different levels in the healthcare organization. Early results show that it has been possible to reduce and sustain the frequency of prosthesis joint infection to below 0.5 % for total hip replacement and well below 1.0 % for total knee replacement. The project has focused on prosthetic joint infection, but it is hoped that the impact will lower the frequency of other healthcare-associated infections also in other types of surgeries.

The second most common AE type in orthopedic care was distended urinary bladder. The results from two theses [22, 23] highlighted this type of AE and it is since 2012 added as a new trigger and AE type in the Swedish handbook. The high frequency of distended urinary bladder may in part be explained by the work to reduce the use of urinary catheters in order to reduce the occurrence of urinary tract infection. The findings point to the importance to implement routines that will lead to both a reduction in the use of urinary catheters, and the frequency of distended urinary bladder.

Patients ≥ 65 years had more admissions with AEs compared to younger patients and this finding concurs with other studies [4, 12, 16, 17, 24, 25]. The mean age in orthopedic care is high and interventions such as surgery are carried out at high ages and on frail patients. Elderly patients are more likely to have more co-morbidities that require more interventions during hospital stay, leading to an increased risk for AEs. Older patients were more often affected by pressure ulcers compared to younger patients, and may also be vulnerable to the effects of healthcare errors, for example, medication errors [25]. Elderly patients have been found to be affected by major AEs to a greater degree than younger patients [26–28].

We found that patients affected by AEs had a longer LOS, which is consistent with other RRR reports [4, 14, 15, 28–30] and may indicate that the orthopedic AEs themselves prolonged LOS.

The use of RRR gives an overview of the incidence, nature, preventability, and consequences of AEs. There is a need of multiple data sources since different data collection methods will complement each other in the work to identify and follow-up improvement areas [31]. However, measurement alone will not lead to an increased patient safety; a systematic quality improvement work based on facts is needed. AEs related to non-operative care have been found to be more frequent than AEs related to surgical techniques, leading to the conclusion that patient safety improvements also need focus on non-operative care processes in surgical care [19, 28]. Two systematic reviews have presented several interventions to reduce the burden of AEs in surgical care such as; improving nurse to patient ratio, team training and communication, use of checklists, and adherence to care pathways [32, 33]. Based on our results, we suggest that every department assesses their specific pattern, and directs their patient safety work accordingly.

### Strengths and limitations

The strengths in our study are the sample size and that the study was performed on a national level with random samples of admissions.

There are several limitations in the present study. One is the method itself, requiring documentation quality in the records, otherwise leading to a risk of underestimation of the numbers of AEs. However, several studies have shown that RRR is a valid method to identify AEs compared to other methods, for example, self reported data such as clinical incident reports [2–4].

Regardless of which RRR method is used, the methodology can be subjective and affected by certain biases such as hindsight bias, which may provide an overestimation, especially of the preventability and severity of an AE. On the other hand, reporting bias and the used follow-up period may lead to an underreporting of AEs. Only AEs identified at the respective hospital were included, leading to those AEs identified and treated in other hospitals or at a general practitioner were missed. Furthermore, only care including an admission was included, which means that strictly outpatient care is not included.

The variability between the hospitals regarding the AE incidence was wide. This is to be expected, since rates are based on cross sectional analyses of small samples allowing large random variation. There could also be other reasons such as that the knowledge and experience of the method varied between and within the review teams. A 1-day education was performed for all review teams, but how the respective reviewer and team interpret the method is unknown, since no inter-rater reliability analysis was performed. All review teams did not include orthopedic surgeons or registered nurses within orthopaedic care. The number of inpatient beds, the case-mix, and the included subspecialties within orthopedics at the respective hospital can vary between the hospitals.

In the national database, no documentation was available regarding if the included admissions were elective or acute, the co-morbidity of the patients, or if the patients underwent surgery or not. These data are only available at the local level.

## Conclusions

We found AEs in 15 % of orthopedic care admissions, healthcare-associated infections and distended urinary bladder being the most common. The majority of AEs was of temporary nature and judged preventable. Systematic and specific patient safety initiatives are needed to reduce the incidence of AEs in orthopedic care.

## Abbreviations

AE: Adverse events
NCC MERP: National Coordinating Council for Medication Error Reporting and Prevention Index
RRR: Retrospective record review
SALAR: Swedish Association of Local Authorities and Regions
